# Body morphology and handgrip strength parameters of the female canoe slalom paddlers

**DOI:** 10.3389/fphys.2024.1343341

**Published:** 2024-02-20

**Authors:** Jan Busta, Jaroslav Hellebrand, Ivana Kinkorová, Andrea Duchoňová, Tereza Hybská, Carmen Costa Sánchez, Matej Vajda

**Affiliations:** ^1^ Department of Swimming, Water and Technical Sports, Faculty of Physical Education and Sport, Charles University in Prague, Prague, Czechia; ^2^ Sport Sciences-Biomedical Department, Faculty of Physical Education and Sport, Charles University in Prague, Prague, Czechia; ^3^ National Institute of Physical Education of Catalunya—INEFC Lleida, Universitat de Lleida, Lleida, Spain; ^4^ Faculty of Physical Education and Sport, Comenius University in Bratislava, Bratislava, Slovakia

**Keywords:** water sport activities, somatotype and body composition, canoeing and kayaking, women and sport, female athlete

## Abstract

**Introduction:** Canoe slalom is a physically very demanding discipline, in which body constitution, body composition, and relative strength are significant factors in high performance. Although anthropometric and strength parameters are relatively well-studied in male athletes, there is a lack of evidence for any conclusions in women. Therefore, the objective of this study was to determine the morphology and upper-limb strength parameters of female canoe slalom paddlers and identify whether morphological differences exist between performance groups.

**Methods:** Altogether, 63 female competitors of the 2023 ICF Canoe Slalom World Cup (*n* = 29) and 2023 ICF Canoe Slalom World Ranking Competition (*n* = 34) in Prague (Czech Republic) were examined with a battery of anthropometric tests, segmental bioimpedance analysis, and handgrip strength test. The athletes were divided into groups according to age and performance: elite athletes belonging to the world top 10 according to the ICF World Ranking (WORLD, *n* = 7), international-level athletes competing during the World Cup (ELITE, *n* = 22), international-level junior athletes competing in the World Ranking Race (JUNIOR, *n* = 17), and other lower performance-level athletes competing in the World Ranking Race (REST, *n* = 17).

**Results:** Female slalom paddlers are, in general, of average body height (∼165 cm), lower body mass (∼60 kg), BMI (∼22 kg/m^2^), and body fat (∼20%) and without exceptional anthropometric dimensions and proportions. However, differences were detected when performance was factored in. Female paddlers belonging to the world TOP 10 have the largest circumferences of arms and forearms, and their somatotype is more mesomorphic, with a lower proportion of total body fat and a higher proportion of muscle mass. In addition, the WORLD group differs significantly in upper-limb strength.

**Discussion:** The results shows the significance of muscular strength and power for canoe slalom athletes and the importance of well-developed musculature in operating the boat in the unstable environment. Being a successful female canoe slalom athlete requires a well-developed musculature, maximizing strength capabilities while maintaining a low body weight through limited hypertrophy of the lower limbs and a low level of body fat.

## Introduction

Canoe slalom is a timed event where competitors navigate a white-water course by passing through a combination of upstream and downstream gates. There are two boat categories for women and men: kayakers (K1) have two paddle blades and are seated in their boat; canoeists (C1) have a single blade and kneel in their boat. Most slalom courses take 80–120 s to complete for the fastest paddlers, depending on the level of competition, difficulty of course, degree of water turbulence, and ability of the other paddlers (Hunter, 2009; Nibali et al., 2011; Vajda and Piatrikova, 2022).

Canoe slalom is a physically very demanding discipline that requires a high level of specific agility (Baláš et al., 2020). This agility is based, among other things, on a high level of fitness, in particular a high level of relative strength, which is in relation to body weight (Busta and Suchý, 2016). In male athletes, physiological differences between performance levels are described—successful male athletes achieve significantly higher levels of relative strength (strength/body weight ratio) and special endurance represented by specific on-water tests (Busta et al., 2018a; Vajda and Piatrikova, 2022) but not in aerobic power tested by treadmill running (Bielik et al., 2021). Therefore, it is not surprising that significant factors in canoe slalom are body size (body measurements and proportions) and body composition (Messias et al., 2021). The elite male athletes are, on average, tall; their body weight is approximately 75 kg and only rarely exceeds 80 kg (Coufalová et al., 2021). They have very well-developed musculature in the trunk and upper limbs, a low body fat percentage, and low muscle volume in the lower limbs (Busta et al., 2018a; Coufalová et al., 2021; Busta et al., 2022). We conclude that anthropometric parameters are very well-studied in men: we know the differences between C1 and K1 paddlers (Coufalová et al., 2021) and also between the highest international performance-level paddlers (including only Olympic and World Championship medalists) and international performance-level paddlers (including remaining World Cup racers) (Busta et al., 2022), national team competitors and other competitors (Busta et al., 2018b), or junior and senior paddlers (Busta and Suchý, 2016). Moreover, we know that the upper-limb strength, which is well represented by the handgrip test (ACSM, 2014), is significantly higher in the world’s most successful athletes compared to other international-level athletes (Busta et al., 2022). However, our understanding of anthropometric, fitness, and physiological dispositions in female athletes is incomplete at best. It is not well-known how different the top performance-level athletes are in terms of body size and body composition because most studies focus on men (Akca and Muniorglu, 2008; Bily et al., 2011; Vedat, 2012; Gao and Zhu, 2023). Studies including women do not usually have a research sample of sufficient size (Krawczyk and Majle, 1994; Coufalová et al., 2021), are older, and do not involve the C1 category (Ridge et al., 2007); participants are not of adult age (Alacid et al., 2012; Okun et al., 2020); or performance groups are not compared. Women, in general, have been neglected in research related to canoeing disciplines, which causes the training regimen not to be based on evidence; hence, this study focuses on female athletes only.

It is evident that in female athletes, it is necessary to investigate all factors influencing the canoe slalom performance. The objective of this study was limited to determine the morphology and handgrip strength of female canoe slalom paddlers and identify whether morphological and strength differences exist between the athletes of different performance levels and age categories. We hypothesized a number of significant differences in body constitution, body composition, and upper-limb strength between performance and age groups that are probably closely related to canoe slalom performance. With the official support of organizers and International Canoe Federation (ICF), over 20 anthropometric measurements, body composition, and handgrip data were obtained from 63 female athletes competing during the 2023 ICF World Cup in June and 2023 ICF World Ranking Race in July (Prague, Czech Republic). This study should provide a better understanding of the body morphology and upper-limb strength of elite female paddlers since the measurements were acquired just days prior to or during the international competition.

## Materials and methods

### Participants

Altogether, 63 female competitors (both boat categories together) of the 2023 ICF Canoe Slalom World Cup (*n* = 29; 33% of the competitors) and 2023 ICF Canoe Slalom World Ranking Competition (*n* = 34; 30% of the competitors) in Prague (Czech Republic) were examined with a battery of anthropometric tests, segmental bioimpedance analysis, and a handgrip strength test. The athletes were divided into groups according to performance-level classification by McKay et al. (2022) and age as follows:

• World-class athletes (WORLD, *n* = 7) competing during the World Cup race and, at the same time, belonging to the world top 10 according to the ICF World Ranking (version 2023-1-X available at www.canoeicf.com);
• Elite athletes (ELITE, *n* = 22), international-level athletes, competing during the World Cup, in which only members of national teams (best three athletes of each nation) are allowed to participate (*n* = 22);
• International-level junior athletes (JUNIOR, *n* = 17) competing in the World Ranking Race and, at the same time, belonging to the national teams (best three junior athletes of each nation);
• National-level athletes (REST, *n* = 17), lower performance-level athletes, competing in the World Ranking Race who are not part of national teams.

The ELITE group was taken as a reference group for comparison because it was the largest and most representative group.

Athletes were contacted and invited to take a part in this study through team officials and prospects distributed over the canoe slalom venue. The criteria for participation in the research were the performance level (given by the possibility to participate in the international race) and age (given by the ICF rules, when over 15 years of age are allowed the start). Participation in the research was voluntary. Only athletes competing during the World Cup and World Ranking Race could take part in this study. Specific rules for measurements were established. All participants have read and signed the informed consent form before measurements. The study was approved by the Ethics Committee at the Faculty of Physical Education and Sport, Charles University in Prague, Czech Republic.

### Data collection

The measurement took place days during the 2023 ICF Canoe Slalom World Cup in Prague (7.—10 June, between 9 a.m. and 5 p.m.) and over two consecutive days during the 2023 ICF Canoe Slalom World Ranking Competition in Prague (28–29 July, between 9 a.m. and 5 p.m.), always in the boathouse gym area. The same assessment procedure was conducted each day to allow for a large sample size and was realized in the same order: anthropometric measurement, next body composition, and then handgrip strength. To eliminate inter-rater variability, all measurements were conducted by experienced examiners from the Faculty of Physical Education and Sport. Each individual examination lasted approximately 20 min. Before the measurement, the athletes answered questions about the boat category and the sport age, which was defined as the period of systematic canoe slalom training.

### Anthropometric measurements and somatotyping

In the data collection of anthropometric parameters, standard methods were followed, and licensed anthropometric instruments were used. Anthropometric measurements were carried out in accordance with standard anthropometric techniques recommended by Norton and Olds (1996), and the standard procedures for each measurement by ISAK (International Society for the Advancement of Kinanthropometry) were followed at all times. Body height was measured and determined to the nearest centimeter on a free-standing medical stadiometer, SECA 220. Body weight was extracted from the body composition analysis of Tanita MC-980 MA. The arm span was measured in the standing position with subjects back facing the wall with outstretched arms, and the value at the furthest fingertips was recorded. The measurement bar was height-adjusted parallel to the ground at the shoulder height. Skinfold measurements were taken using a Harpenden skinfold caliper (Skinfold HSC 4) at the following sites: triceps, subscapular, suprailiac, thigh, and calf. Body circumferences were measured using the soft metric tape. Epicondyle widths (humerus and femur) were measured using the medical T-520 modified thoracometer. All unilateral measurements were performed on the right side of the body. Somatotypes were calculated according to Carter and Heath (1990).

### Body composition

Body composition, including body weight, muscle mass, and body fat contribution, was evaluated using the multi-frequency device Tanita MC-980 MA. Participants were asked not to eat for 2 h and drink 1 h before the measurement. Testing was performed in underwear only in a standing position with arms extended down.

### Grip strength

The handgrip isometric strength was assessed with a conventional dynamometer (Takei TKKK 5401, Takei Scientific Instruments, Tokyo, Japan). In a sitting position, the paddlers grasped the hand dynamometer with an elbow in full extension, arm near the body, and gradually applied maximal pressure for at least 2 s. First, three trials with the right arm and then three trials with the left arm were examined. The best of three consecutive trials was considered for data analysis. A 30-s recovery was allowed between trials. When applying the grip force, the stretched hand was not allowed to touch any part of the body. The adjustable part of the handle was set to reach the first phalanx of the ring finger. The values were calculated as the maximal force production (kgf) and the relative maximal force, where the measured handgrip force was divided by the body weight in kilograms (kgf/kg).

### Data analysis

In the basic descriptive statistics, mean and standard deviation are used. To find out the differences between the groups, the independent Student’s t-test is used. Statistical significance was set at *p* < 0.05. Cohen’s d was used to find practical differences. All statistical calculations were performed using IBM SPSS for Windows (version 24, Chicago, Illinois, United States). Effect sizes were classified as trivial (0–0.2), small (0.2–0.6), moderate (0.6–1.2), large (1.2–2.0), and very large (>2.0) (Hopkins, 2006). A difference between groups of more than 5% was also judged to be practically significant. The ELITE group was taken as a reference group. In addition to the tabular form, the comparison between the WORLD and JUNIOR group was prepared. The radar graphs with markers have been calculated for ELITE and WORLD as 10-(0.5×d) for ELITE and 10 + (0.5 × d) for JUNIOR.

## Results

Table 1 shows the comparison between elite international-level athletes competing during the World Cup (ELITE; *n* = 22), world class athletes belonging to the (WORLD; *n* = 7) group, international-level junior athletes competing in the World Ranking Race (JUNIOR; *n* = 17), and other lower performance-level athletes competing in the World Ranking Race (REST; *n* = 17).

**TABLE 1:** Characteristics of the performance groups of the female canoe slalom athletes.

Variable	ELITE (*n* = 22)	WORLD (*n* = 7)	JUNIOR (*n* = 17)	REST (*n* = 17)
Age (years)	23.2 ± 4.3	24.6 ± 3.0	16.4 ± 1.2	19.1 ± 5.8
Sport age (years)	11.7 ± 3.9	15.4 ± 2.8	8.3 ± 3.0	6.9 ± 4.4
Body mass (kg)	60.5 ± 6.4	62.8 ± 7.0	59.4 ± 7.4	60.1 ± 7.7
Height (cm)	165.6 ± 7.5	167.4 ± 5.5	166.6 ± 5.2	163.3 ± 5.2
Body mass index (kg/m^2^)	22.0 ± 1.4	22.4 ± 1.7	21.4 ± 1.8	22.7 ± 2.4
Sitting height (cm)	86.9 ± 3.2	88.9 ± 2.3	88.8 ± 3.1	85.9 ± 3.2
Arm span (cm)	166.3 ± 9.0	168.0 ± 6.4	165.6 ± 7.7	163.0 ± 6.3
Sitting height/body height (%)	0.53 ± 0.01	0.53 ± 0.01	0.53 ± 0.01	0.53 ± 0.01
Arm span/body height (%)	1.0 ± 0.02	1.0 ± 0.02	0.99 ± 0.02	1.0 ± 0.02
Humerus breadth (cm)	6.1 ± 0.4	6.3 ± 0.2	6.1 ± 0.4	5.7 ± 0.9
Femur breadth (cm)	8.9 ± 0.5	9.2 ± 0.3	9.0 ± 0.5	8.9 ± 1.2
Shoulder breadth (cm)	41.2 ± 2.7	41.7 ± 5.9	40.5 ± 2.2	39.6 ± 2.9
Forearm girth (cm)	24.9 ± 1.2	25.6 ± 1.4	24.9 ± 4.7	25.2 ± 1.5
Flexed arm girth (cm)	30.6 ± 2.0	31.1 ± 1.4	29.5 ± 2.0	30.0 ± 2.5
Thigh girth (cm)	49.8 ± 3.1	50.6 ± 3.7	49.0 ± 3.6	49.8 ± 3.2
Calf girth (cm)	34.5 ± 1.8	35.3 ± 2.7	34.6 ± 2.1	34.2 ± 3.7
Sum of five skinfolds (mm)	47.3 ± 9.1	41.7 ± 5.9	50.5 ± 14.2	55.0 ± 10.0
Endomorphy	2.7 ± 0.6	2.5 ± 0.5	2.9 ± 0.7	3.3 ± 0.8
Mesomorphy	4.4 ± 0.9	4.8 ± 1.0	4.0 ± 0.8	4.2 ± 1.7
Ectomorphy	2.4 ± 0.8	2.2 ± 0.9	2.8 ± 0.8	2.1 ± 1.0
Body fat (%)	18.6 ± 3.8	17.9 ± 3.6	22.6 ± 2.7	22.9 ± 3.9
Muscle mass (kg)	46.6 ± 3.8	48.8 ± 4.1	43.6 ± 5.2	44.2 ± 4.5
Total body water (%)	59.4 ± 2.9	57.8 ± 7.8	56.6 ± 1.9	56.8 ± 3.1
Extracellular water/total body water (%)	37.1 ± 1.1	36.0 ± 0.7	36.7 ± 1.1	37.0 ± 0.5
Handgrip right hand (kgf)	33.5 ± 6.0	39.0 ± 3.7	32.0 ± 3.9	32.1 ± 5.2
Handgrip right hand relativized (kgf/kg)	0.55 ± 0.07	0.62 ± 0.07	0.55 ± 0.09	0.54 ± 0.08
Handgrip left hand (kgf)	33.3 ± 5.3	38.0 ± 5.2	31.0 ± 4.1	31.8 ± 6.3
Handgrip left hand relativized (kgf/kg)	0.55 ± 0.06	0.61 ± 0.10	0.53 ± 0.09	0.53 ± 0.10

Table 2 shows the assessment of the statistical and substantive significance of the differences between the groups. The ELITE group differs significantly from the WORLD group in the following parameters: sport age (11.7 ± 3.9 vs. 15.4 ± 2.8 years; *p* = 0.03, d = 1.02) by 31.6%, sum of five skinfolds (47.3 ± 9.1 vs. 41.7 ± 5.9 mm; d = 0.73) by 11.8%, ECW/TBW (37.1 ± 1.1 vs. 36.0 ± 0.7; *p* = 0.02, d = 1.02) by 3%, right-hand handgrip (33.5 ± 6.0 vs. 39.0 ± 3.7 kgf; *p* = 0.01, d = 1.10) by 16.4%, relativized right-hand handgrip (0.55 ± 0.07 vs. 0.62 ± 0.07 kgf/kg; *p* = 0.02, d = 1) by 12.7%, left-hand handgrip (33.3 ± 5.3 vs. 38.0 ± 5.2 kgf; *p* = 0.05, d = 0.89) by 13.4%, and relativized left-hand handgrip (0.55 ± 0.06 vs. 0.61 ± 0.10 kgf/kg; *p* = 0.05, d = 0.72) by 10.9%.

**TABLE 2:** Statistic and practical differences between the performance groups.

Variable	Difference								
	ELITE/WORLD			ELITE/JUNIOR			ELITE/REST		
	p	d	%	p	d	%	p	d	%
Age (years)	0.44	0.34	6.0	**0.00**	**2.03**	**−29.3**	**0.02**	**0.82**	**−17.7**
Sport age (years)	**0.03**	**1.02**	**31.6**	**0.01**	**0.98**	**−29.1**	**0.00**	**1.16**	**−41**
Body mass (kg)	0.43	0.34	3.8	0.62	0.16	−1.2	0.85	0.06	−0.7
Height (cm)	0.56	0.25	1.1	0.64	0.15	−0.6	0.30	0.34	−1.4
Body mass index (kg/m2)	0.59	0.23	1.8	0.19	0.42	−2.7	0.32	0.32	3.2
Sitting height (cm)	0.13	0.71	2.3	0.07	0.60	2.2	0.34	0.31	−1.1
Arm span (cm)	0.66	0.19	1.0	0.80	0.08	0.4	0.20	0.42	−2.0
Sitting height/body height (%)	0.30	0	0	0.08	0	0	0.85	0	0
Arm span/body height (%)	0.92	0	0	0.16	0	0	0.30	0	0
Humerus breadth (cm)	0.24	0.51	3.3	0.81	0.07	3.3	0.06	0.63	−6.5
Femur breadth (cm)	0.21	0.56	3.4	0.70	0.12	4.5	0.97	0.01	0
Shoulder breadth (cm)	0.49	0.30	1.2	0.37	0.29	−1.2	0.08	0.59	−3.9
Forearm girth (cm)	0.16	0.62	2.8	0.93	0.02	0	0.42	0.26	1.2
Flexed arm girth (cm)	0.56	0.25	1.6	0.10	0.58	−3.6	0.42	0.26	−2.0
Thigh girth (cm)	0.58	0.24	1.6	0.47	0.23	−1.6	0.97	0.01	0
Calf girth (cm)	0.36	0.40	2.3	0.82	0.07	0.3	0.80	0.08	−0.9
Sum of five skinfolds (mm)	0.14	**0.73**	**−11.8**	0.40	0.26	**6.8**	**0.02**	**0.80**	**16.3**
Endomorphy	0.40	0.36	**−7.4**	0.21	0.30	7.4	**0.01**	**0.84**	**22.2**
Mesomorphy	0.29	0.42	**9.1**	0.22	0.47	9.1	0.69	0.14	−4.5
Ectomorphy	0.60	0.23	**−8.3**	0.13	0.5	16.7	0.35	0.33	12.5
Body fat (%)	0.67	0.18	−3.8	**0.00**	**1.16**	**21.5**	**0.00**	**1.11**	**23.1**
Muscle mass (kg)	0.20	0.56	4.7	**0.04**	0.67	**−6.4**	0.07	0.59	−5.1
Total body water (%)	0.41	0.36	−2.7	**0.01**	**1.09**	4.7	**0.00**	**0.86**	−4.4
Extracellular water/total body water (%)	**0.02**	**1.02**	−3.0	0.53	0.38	1.1	0.76	0.14	−0.3
Handgrip right hand (kgf)	**0.01**	**1.10**	**16.4**	0.25	0.29	4.5	0.97	0.24	−4.2
Handgrip right hand relativized (kgf/kg)	**0.02**	**1**	**12.7**	0.97	0	0	0.73	0.13	−1.8
Handgrip left hand (kgf)	**0.05**	**0.89**	**13.4**	0.18	0.48	**−6**	0.29	0.25	−4.5
Handgrip left hand relativized (kgf/kg)	**0.05**	**0.72**	**10.9**	0.38	0.26	3.6	0.51	0.24	−3.6

Differences that can be considered statistically or substantively significant have been marked in bold.

Significant differences were also found between the ELITE group and the JUNIOR group in the following parameters: age (23.2 ± 4.3 vs. 16.4 ± 1.2 years; *p* = 0.00, d = 2.03) by 29.3%, sport age (11.7 ± 3.9 vs. 8.3 ± 3 years; *p* = 0.01, d = 0.98) by 29.1%, sum of five skinfolds (47.3 ± 9.1 vs. 50.5 ± 14.2 mm) by 6.8%, body fat (18.6% ± 3.8% vs. 22.6% ± 2.7%; *p* = 0.00, d = 1.16) by 21.5%, muscle mass (46.6 ± 3.8 vs. 43.6 ± 5.2 kg; *p* = 0.04) by 6.4%, and total body water (59.4% ± 2.9% vs. 56.6% ± 1.9%; *p* = 0.01, d = 1.06) by 4.7%.

Between ELITE and REST groups were found the following differences: age (23.2 ± 4.3 vs. 19.1 ± 5.8 years; *p* = 0.02, d = 0.82) by 17.7%, sport age (11.7 ± 3.9 vs. 6.9 ± 4.4 years; *p* = 0.00; d = 1.16) by 41%, body fat (18.6% ± 3.8% vs. 22.9% ± 3.9%) by 23.1%, sum of five skinfolds (47.3 ± 9.1 vs. 55.0 ± 10 mm; *p* = 0.02, d = 0.80) by 16.3%, and total body water (59.4% ± 2.9% vs. 56.8% ± 3.1%; *p* = 0.00, d = 0.86) by 4.4%. A significant difference was also found in the parameter of endomorphy (2.7 ± 0.6 vs. 3.3 ± 0.8; *p* = 0.01, d = 0.84) by 22.2%.

In addition, several significant differences were observed between WORLD and JUNIOR groups in following differences: age (24.6 ± 3.0 vs. 16.4 ± 1.2 years; *p* = 0.00, d = 4.4) by 33.3%, sport age (15.4 ± 2.8 vs. 8.3 ± 3 years; *p* = 0.00, d = 2.5) by 46.1%, right-hand handgrip (39.0 ± 3.7 vs. 32.0 ± 3.9 kgf; *p* = 0.02, d = 1.5) by 18%, relativized right-hand handgrip (0.62 ± 0.07 vs. 0.55 ± 0.09 kgf/kg; *p* = 0.00; d = 0.86) by 11.3%, left-hand handgrip (38.0 ± 5.2 vs. 31.0 ± 4.1 kgf; *p* = 0.03, d = 1.5) by 18.4%, relativized left-hand handgrip (0.61 ± 0.10 vs. 0.53 ± 0.09 kgf/kg; *p* = 0.03, d = 0.84) by 13.1%, body fat (17.9% ± 3.6% vs. 22.6% ± 2.7%; *p* = 0.00, d = 1.6) by 26.3%, and muscle mass (48.8 ± 4.1 vs. 43.6 ± 5.2 kg; *p* = 0.02, d = 1.1) by 10.7%.

**FIGURE 1:**
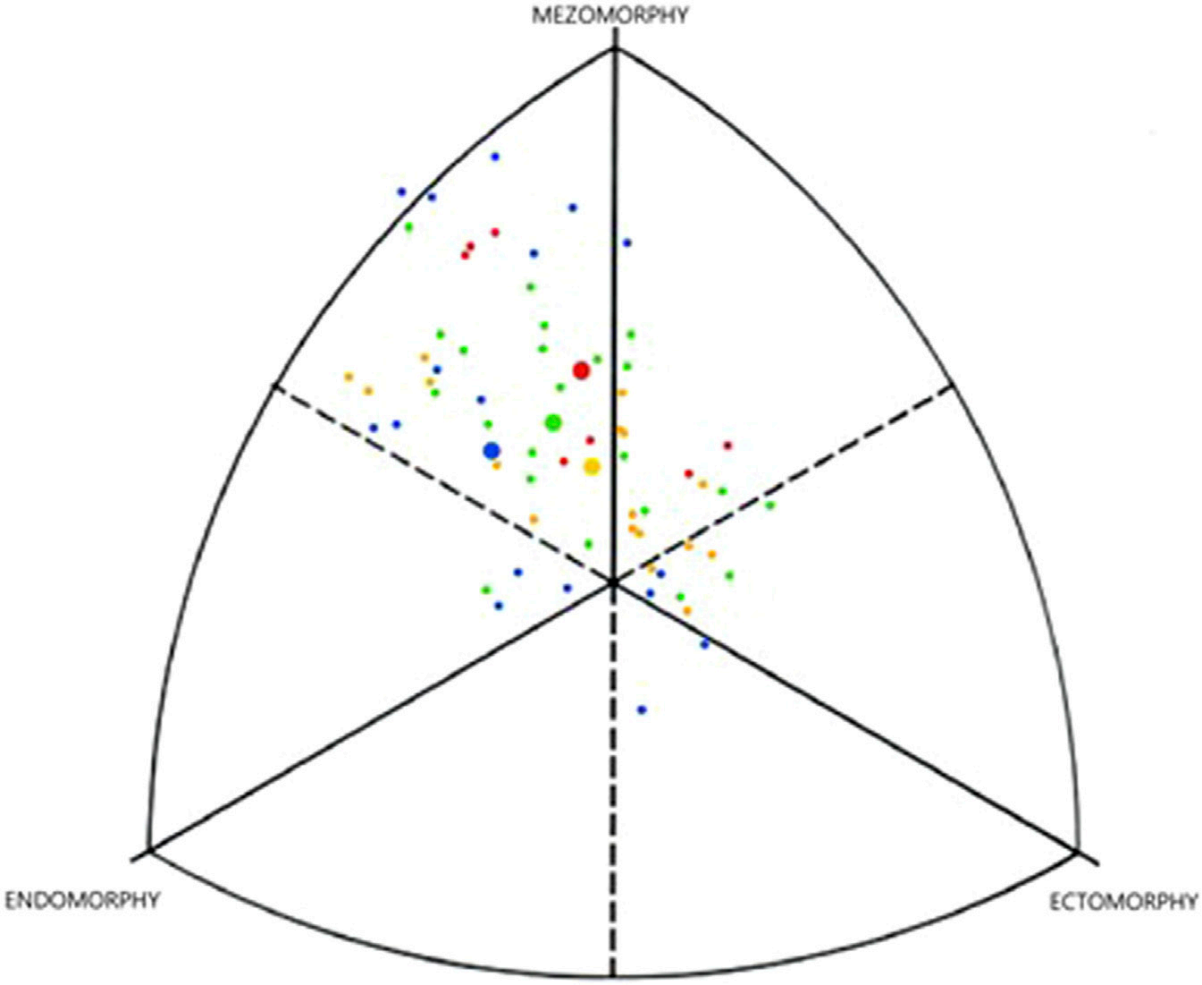
Individual and average somatotypes of the canoe slalom female competitors. 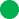
average ELITE
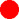
average WORLD
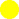
average JUNIOR
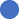
average REST.

**FIGURE 2:**
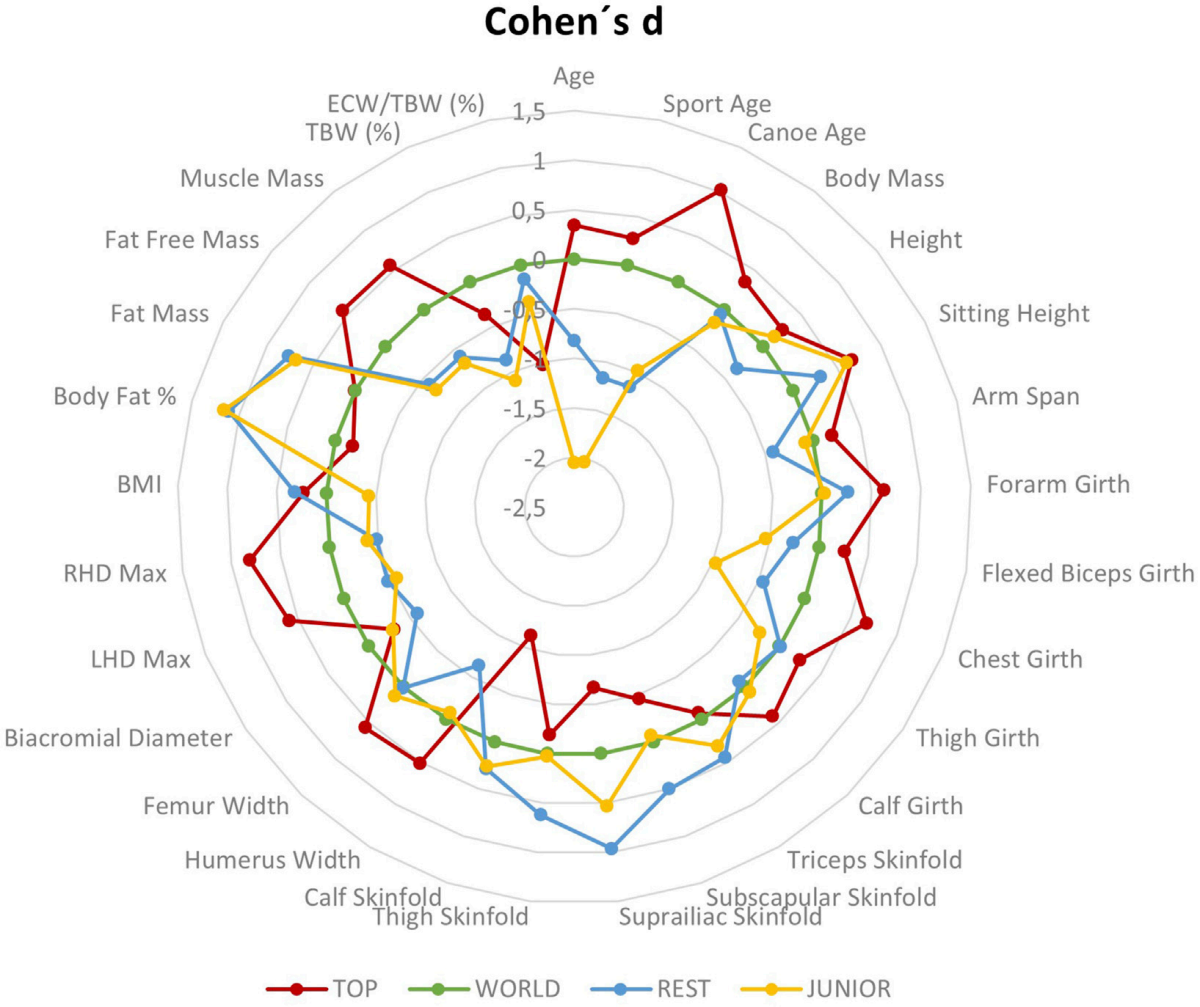
Radar graph with markers to present intergroup differences in female paddlers.

In Figure 1, we can observe a considerable variance of the evaluated somatotypes. However, distinctly mesomorphic somatotypes occur almost only in ELITE and WORLD groups. In addition, the average somatotype of these groups is the most mesomorphic. The radar graph (Figure 2) presents graphically differences between defined performance groups.

## Discussion

Numerous inherent anthropometric, physical, and physiological factors could potentially impact the performance capacity of WORLD/ELITE athletes. The research was limited by methods that did not physically and mentally stress the athletes just before the race. Testing of physiological/fitness performance parameters prior to competition is not feasible, but access to a research sample of female athletes from around the world is equally problematic outside of the competition period. Of course, participation on a voluntary basis and size of the research sample are also limited, as is the fact we did not reflect hormonal status and/or puberty phase, but this is quite common for such studies. Therefore, the aim of this study was limited to determine the morphology of female canoe slalom paddlers and identify whether morphology and handgrip strength differences existed between performance groups. We were able to accurately describe the anthropometric and strength parameters of female performance athletes in canoe slalom and describe the differences between the defined performance groups. Based on the previous research (Coufalová et al., 2021), we did not reflect boat category (kayak—K1, canoe—C1) because no significant differences in the monitored parameters between the competitors of the given categories were found before.

Canoe slalom, being a sport that requires considerable technical skills, is often dominated by older and more experienced athletes. The average age of female participants in the 2000 Sydney Olympics in canoe slalom was 26.3 ± 4.8 (20–35) (Ridge et al., 2007). An average sporting age of 15.4 ± 2.8 years is notable in world class athletes belonging to the WORLD group. WORLD group female athletes have been participating in canoe slalom for a third longer than other World Cup participants belonging to the ELITE group (*p* = 0.03, d = 1.02), and the difference is even higher compared to that of junior female competitors (JUNIOR) and competitors of a lower performance level (REST).

When comparing our findings from athletes belonging to the ELITE group with the previous research on female slalom 2000 Olympian paddlers (Ridge et al., 2007), the somatotype components (2.7–4.4–2.4 vs. 2.4–4.4–2.6) have remained relatively consistent over 2 decades. The WORLD group was distinguished by a higher proportion of mesomorphic components in their somatotype (2.5–4.8–2.2), and their somatotype was more distinctly mesomorphic.

Female slalom paddlers are, in general, of average body height (Garcia and Quintana-Domeque, 2007), and according to Ridge et al. (2007), female slalom paddlers are not characterized by any extraordinary dimensions and proportions. The female athletes of the ELITE group were of very similar body height (165.6 vs. 168.0 cm) and body weight (60.5 ± 6.4 vs. 59.0 ± 4.5 kg) like the K1 2000 Olympian paddlers (Ridge et al., 2007) or female 2018 European championship competitors (58.8 ± 4.6 kg; 164.2 ± 5.4 cm) (Coufalová et al., 2021). Similar mean values, as in our study were found, when compared with elite female canoe polo athletes (Sheykhlouvand and Forbes, 2018), presenting a mean of weight 61.4 ± 7.1 kg and height 166.9 ± 5.2 cm. In comparison with female canoe sprint paddlers (Ackland et al., 2003), canoe slalom athletes present similar body height but notable difference in their body weight (62.8 ± 7.0 vs. 67.3 ± 5.9 kg). Female rowers are not only heavier (73.4 ± 5.2 kg) but also taller (176.7 ± 6.4 cm) (Busta et al., 2023). The female competitors of the defined groups do not differ significantly from each other in terms of body height and body weight; with regards to body height, they do not differ from the general population of the respective age (Garcia and Quintana-Domeque, 2007). However, female athletes of higher performance levels have a higher proportion of muscle mass and lower total body fat at similar body weights. These differences were observed by both body measurement methods, i.e., caliperation and bioimpedance. The WORLD group differs significantly from the ELITE group in the sum of five skinfolds (41.7 ± 5.9 vs. 47.3 ± 9.1 mm; d = 0.73) by 11.8%, and similar is the difference between the WORLD group and 2018 European Championship competitors (53.0 ± 13.0 mm) (Coufalová et al., 2021). Therefore, it can be assumed that the lower body fat percentage relates to better performance.

World-class female paddlers belonging to the WORLD group have the largest arm and forearm circumferences; their somatotype is more mesomorphic, with a lower proportion of total body fat. In addition, the WORLD group differs significantly from the ELITE group in the upper-limb strength by 16.4% for the right hand (39.0 ± 3.7 vs. 33.5 ± 6.0 kgf; *p* = 0.01; d = 1.10) and 13.4% for the left hand (38.0 ± 5.2 vs. 33.3 ± 5.3 kgf; *p* = 0.05; d = 0.89). The handgrip strength of athletes belonging to the WORLD group was superior in comparison to 30 female athletes competing at a United States Powerlifting Association (USPA) powerlifting meet in Salt Lake City, who presented a mean of 32.0 ± 7.1 kgf at a body weight of 82.8 ± 27.8 kg in the highest trial (Suazo and Debeliso, 2021). The handgrip strength of WORLD-level canoe slalom athletes is similar to that of open-category female rowers (right hand: 40.8 ± 6.7; left hand: 40.5 ± 7.6 kgf), which weighs approximately 15 kg more (76.4 ± 5.5 kg), while in terms of fat mass in body composition, female kayakers are more similar to lightweight rowers (15.0% ± 3.1%) (Busta et al., 2023).

The differences in upper-limb strength and body composition described above are related not only to the differences in sport age itself but also to the increased volume and intensity of the training process and indirectly indicate a higher level of training. All the differences indicate that being a successful canoe slalom athlete requires a well-developed musculature, maximizing strength capabilities while maintaining a low body weight through controlled hypertrophy of the lower limbs, thereby maintaining a low level of body fat and regular hard training on the water and in the gym. The differences are conditioned by the increased volume and intensity of the training and the duration of the specific training itself (WORLD group had significantly highest sport age: 15.4 ± 2.8 years). Essentially, the same was concluded by earlier studies conducted on male athletes (Busta et al., 2018a; Coufalová et al., 2021; Busta et al., 2022).

## Conclusion

Based on the measurement of 63 female athletes during the 2023 ICF World Cup and World Ranking Competition, we can offer the following conclusions: female slalom paddlers are, in general, of average body height (∼165 cm), lower body mass (∼60 kg), BMI (∼22 kg/m2), and body fat (∼20%), and are not characterized by any extraordinary dimensions and proportions. However, differences were detected when performance was factored in. World-class female paddlers belonging to the WORLD group have a more mesomorphic somatotype with a lower proportion of total body fat. In addition, the WORLD group differs significantly in upper-limb strength. This shows the significance of muscular strength and power for canoe slalom athletes and the importance of well-developed musculature when operating the boat in an unstable environment. Being a successful female canoe slalom athlete requires a well-developed musculature, maximizing strength capabilities while maintaining a low body weight through a low level of body fat. The findings emphasize the specific morphological and strength characteristics that contribute to the success of female canoe slalom athletes at the world stage, accentuating the importance of strength and consistent somatotype patterns in navigating challenging whitewater courses. To maximize performance in canoe slalom, from a morpho-physiological perspective, it is necessary to maintain both a low body mass (∼60 kg) and a high level of strength. Therefore, to achieve world-class performance, it is necessary to reduce % body fat (∼18%) while maintaining/increasing muscle mass and strength.

## Data Availability

The original contributions presented in the study are included in the article/Supplementary Materials, further inquiries can be directed to the corresponding author.
